# CO2 Laser Popularity in Germany: A Five-Year Google Trends Analysis (2020‐2025)

**DOI:** 10.2196/77651

**Published:** 2026-04-21

**Authors:** Michael Constantin Kirchberger, Andreas Eisenried

**Affiliations:** 1 Lehrstuhl Dermatologie Friedrich-Alexander-Universität Erlangen-Nürnberg, Ulmenweg 18, Erlangen, 91054, Germany, 49 9121-853500; 2 Lehrstuhl Anästhesiologie Friedrich-Alexander-Universität Erlangen-Nürnberg, Erlangen, Germany

**Keywords:** fractional CO₂ laser, infodemiology, google trends, aesthetic dermatology, Germany

## Abstract

**Background:**

Fractional carbon dioxide (CO₂) laser resurfacing is widely used for the treatment of scars and photoaging. In recent years, public interest in minimally invasive esthetic procedures has grown, influenced by social media exposure and changing beauty norms. However, data quantifying population-level attention to CO₂ laser treatments in Germany are limited.

**Objective:**

This study aimed to assess the long-term trajectory and seasonal patterns of public information-seeking behavior regarding fractional CO₂ laser treatments in Germany from January 2020 to December 2025 using Google Trends data.

**Methods:**

Monthly normalized search volume (NSV) for the category “Health” and the term “CO2 laser” was retrieved for the period January 2020 to December 2025. Seasonal-Trend decomposition (STL) using LOESS (locally estimated scatterplot smoothing) was applied to isolate the long-term trend from seasonal fluctuations. The significance of the upward trajectory was assessed using linear regression on the extracted trend component, and seasonal differences were evaluated via the seasonal component amplitude.

**Results:**

Public interest in CO₂ lasers increased significantly, with the annual mean NSV rising from 15.0 in 2020 to 68.1 in 2025. Regression analysis of the STL trend component revealed a steady, statistically significant monthly increase (slope=0.87 NSV/month; 95% CI 0.83‐0.92; *P*<.001). Furthermore, a robust seasonal pattern was identified (*P*<.001), with search interest consistently peaking in winter (January mean SD 13.8) and reaching a nadir during the summer months (August SD=–14.4).

**Conclusions:**

Digital information-seeking behavior regarding fractional CO₂ laser treatments in Germany increased by 354% over the past six years, accompanied by consistent, clinically relevant seasonal peaks in winter. These findings reflect broader shifts in esthetic awareness. The identified temporal patterns provide valuable insights for timing educational messaging, managing patient inquiries, and addressing safety considerations in esthetic medicine.

## Introduction

Fractional carbon dioxide (CO₂) laser therapy has become a cornerstone in dermatological and esthetic medicine, particularly for the treatment of scars and skin aging. This modality combines the efficacy of traditional ablative laser techniques with the benefits of fractional technology, resulting in significant skin remodeling with reduced downtime. The fractional approach delivers precise microthermal zones, promoting collagen production and skin rejuvenation while preserving surrounding tissue, thereby minimizing recovery time and adverse effects [1]. Clinical studies have demonstrated the effectiveness of fractional CO₂ lasers in improving the appearance of surgical scars, acne scars, and photoaged skin, with high patient satisfaction rates [1-3].

The increasing demand for minimally invasive cosmetic procedures has been fueled by advancements in laser technology and a growing emphasis on esthetic self-care. Social media platforms have played a significant role in popularizing these treatments, often showcasing dramatic before-and-after results and promoting the benefits of fractional CO₂ laser therapy. This widespread exposure has led to heightened public interest and a surge in the number of individuals seeking information about such treatments [4,5].

Understanding public interest and behavior regarding fractional CO₂ laser treatments is essential for health care providers and policymakers. Google Trends, a tool that analyzes the popularity of search queries over time, has emerged as a valuable resource in health research for gauging public interest and information-seeking behavior. This methodology is rooted in the conceptual framework of infodemiology, defined by Eysenbach [6] as the science of distribution and determinants of information in an electronic medium, specifically with the aim to inform public health and policy. Studies have used Google Trends data to monitor public awareness and interest in various health topics, including mental health, infectious diseases, and cosmetic procedures. The tool’s ability to provide real-time, population-level data makes it particularly useful for tracking trends and informing public health strategies [7-10].

In this study, we aim to analyze public information-seeking behavior regarding fractional CO₂ laser treatments in Germany from 2020 to 2025. Using Google Trends data filtered by the “Health” category and Seasonal-Trend decomposition, we isolate long-term growth from recurring seasonal fluctuations. This analysis is highly timely, as digital curiosity for minimally invasive esthetic procedures surges, regulatory frameworks for medical laser operators face intense professional scrutiny. Understanding these search dynamics provides clinicians and policymakers with critical insights to anticipate patient inquiries, optimize public health education, and mitigate safety risks associated with improper device use.

## Methods

### Data Retrieval and Ethics

Public interest data for the search term “CO2 laser” in Germany were retrieved using Google Trends. To ensure dermatological relevance and exclude industrial applications, the query was restricted to the “Health” category (Category ID: 45). The study period covered six full calendar years, from January 1, 2020, to December 31, 2025. As the dataset consists of aggregated and anonymized search volumes, the study did not involve human subjects and was exempt from institutional review board approval.

### Statistical Analysis

To address the autocorrelation inherent in time-series data and isolate underlying patterns, a Seasonal-Trend decomposition (STL) using LOESS (locally estimated scatterplot smoothing) was applied. This method decomposed the monthly normalized search volume (NSV) into three distinct components: the long-term trend, seasonal variation, and residual noise. The long-term trajectory was evaluated using ordinary least squares linear regression performed on the extracted trend component to calculate the monthly rate of change and its 95% CI. Seasonal variation was quantified by averaging the seasonal component across the six-year period to identify recurring monthly peaks and troughs. All statistical tests were two-tailed, with a significance threshold of α=.05. For regional analysis, the “Interest by Region” output was exported as a single dataset for the entire period (2020‐2025) to ensure consistent normalization across all 16 federal states.

All analyses and visualizations were conducted using Python (version 3.11). Data processing was performed with Pandas and NumPy. Time-series decomposition was executed using the Statsmodels library. Statistical regressions were calculated via SciPy. For geospatial visualization, a high-resolution GeoJSON file of German state borders was merged with the search data using GeoPandas. Regional interest was represented as a choropleth map using the *Matplotlib* library, with federal states labeled using official two-letter abbreviations.

### Ethical Considerations

This study used publicly accessible, fully anonymized, and aggregated search volume data provided by Google Trends. As the research did not involve human subjects, clinical interventions, or the collection of any personally identifiable information, institutional review board approval and informed consent were not required in accordance with local and institutional guidelines.

## Results

### Trends and Seasonal Variation

**Figure 1.**
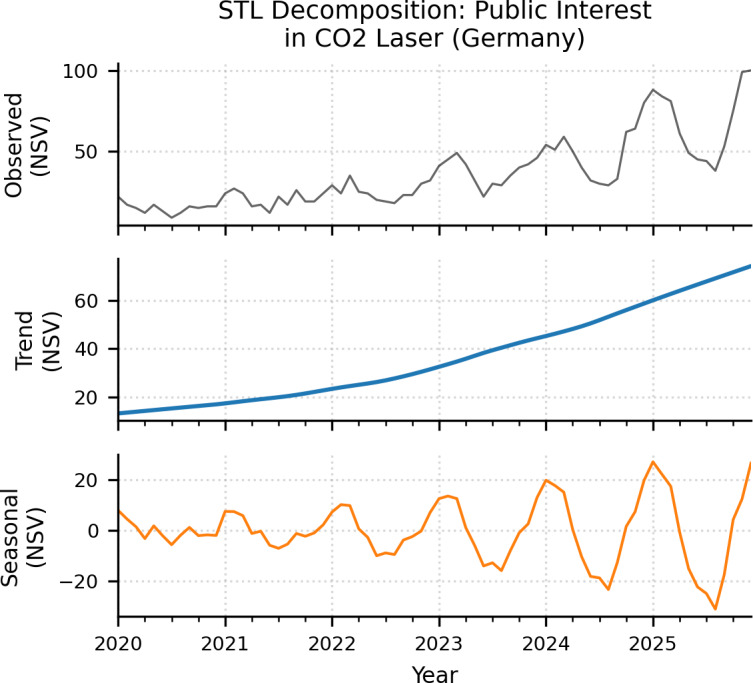
Long-term trend of public interest in CO₂ laser treatments in Germany (2020‐2025). The graph illustrates the isolated trend component extracted via Seasonal-Trend decomposition using locally estimated scatterplot smoothing from monthly Google Trends data (filtered by the “health” category). The analysis reveals a sustained 354% increase in digital information-seeking behavior, with the annual mean normalized search volume (NSV) rising from 15.0 in 2020 to 68.1 in 2025. Linear regression of this isolated component confirms a highly significant upward trajectory over the 72-month period (slope=0.87 NSV/month; 95% CI 0.83‐0.92; *P*<.001).

The longitudinal analysis of search interest for the search term “CO2 laser” in Germany—filtered by the “Health” category to ensure medical relevance reveals a significant and sustained upward trajectory (Figure 1). By applying STL LOESS, the underlying long-term trend was isolated from seasonal noise and residuals, confirming that the growth is not merely a byproduct of transient spikes.

During the initial phase of the study (2020‐2021), the trend component remained at a stable baseline, with annual mean NSV values increasing from 15.0 to 20.6. A more pronounced transition occurred in mid-2022, marking the beginning of a continuous growth phase that reached its peak in 2025 with an annual mean of 68.1 NSV. This represents a total increase in sustained public interest of 354% compared to the 2020 baseline. Linear regression analysis of the isolated trend component confirmed the high statistical significance of this growth (Slope=0.87 NSV/month; 95% CI 0.83‐0.92; R^2^=.96; *P*<.001).

**Figure 2.**
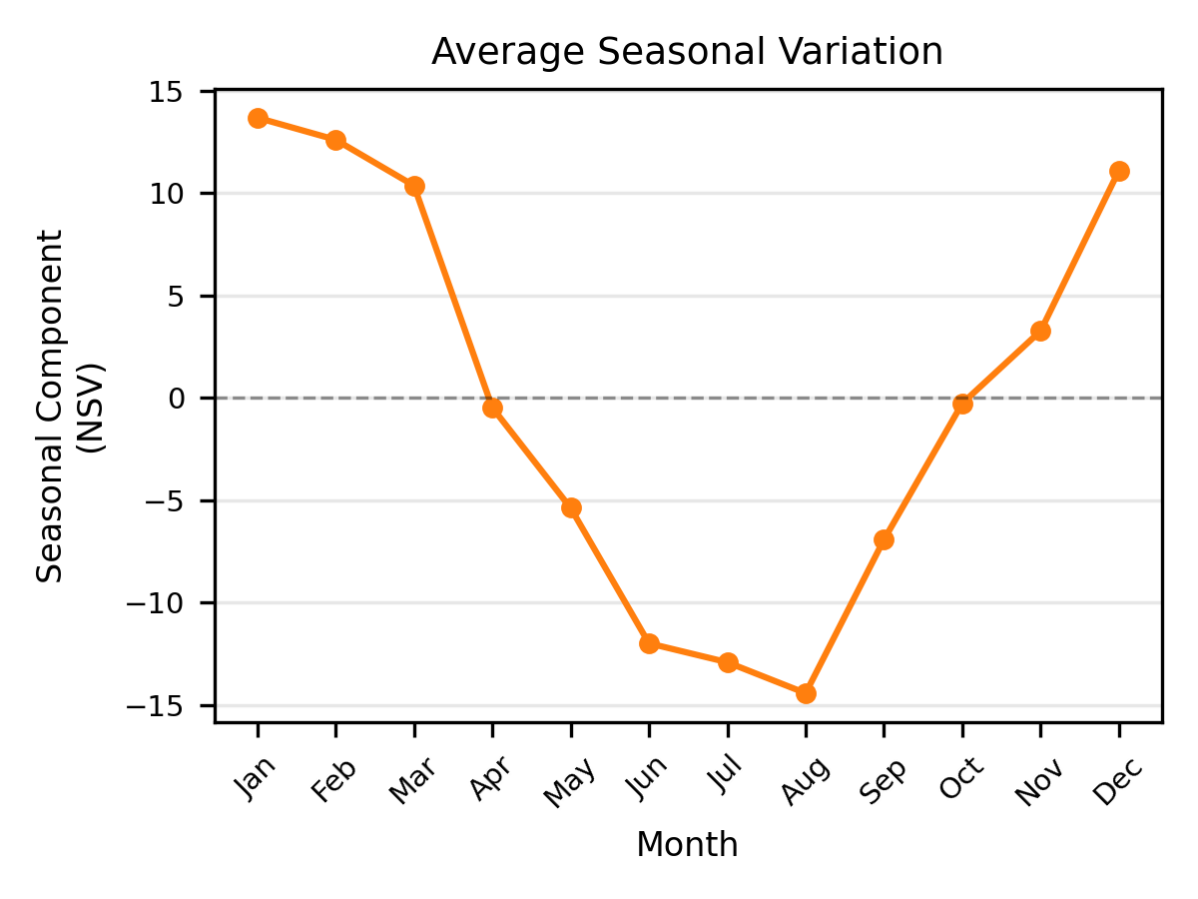
Seasonal variation in search interest for CO₂ laser treatments. The chart displays the isolated seasonal component derived from the STL decomposition, representing the average monthly deviation from the underlying long-term trend. A robust, recurring annual cycle is evident (*P*<.001), with public interest consistently peaking during the winter months, reaching its zenith in January (+13.8 NSV units). Conversely, search activity drops to a predictable nadir during the summer, reaching its lowest point in August (–14.4 NSV units). This cyclical rhythm closely aligns with clinical recommendations to avoid ablative laser procedures during periods of high environmental UV radiation.

Complementing this long-term growth, a robust and recurring seasonal rhythm was identified. While Figure 1 (bottom panel) shows the consistency of these waves over time, Figure 2 illustrates the averaged seasonal deviations to highlight the typical annual cycle. Public interest follows a predictable pattern characterized by a “winter peak” and a “summer nadir.” Information-seeking behavior typically reaches its zenith in January (+13.8 NSV units) and February (+12.6 NSV units).

In contrast, interest consistently declines as annual UV radiation increases, reaching its lowest levels in August (–14.4 NSV units). This seasonal component remained highly consistent across the six-year period, with statistical analysis confirming that these monthly variations are non-random (*P*<.001), likely reflecting clinical recommendations to avoid laser treatments during periods of high solar exposure.

### Yearly Growth

**Figure 3.**
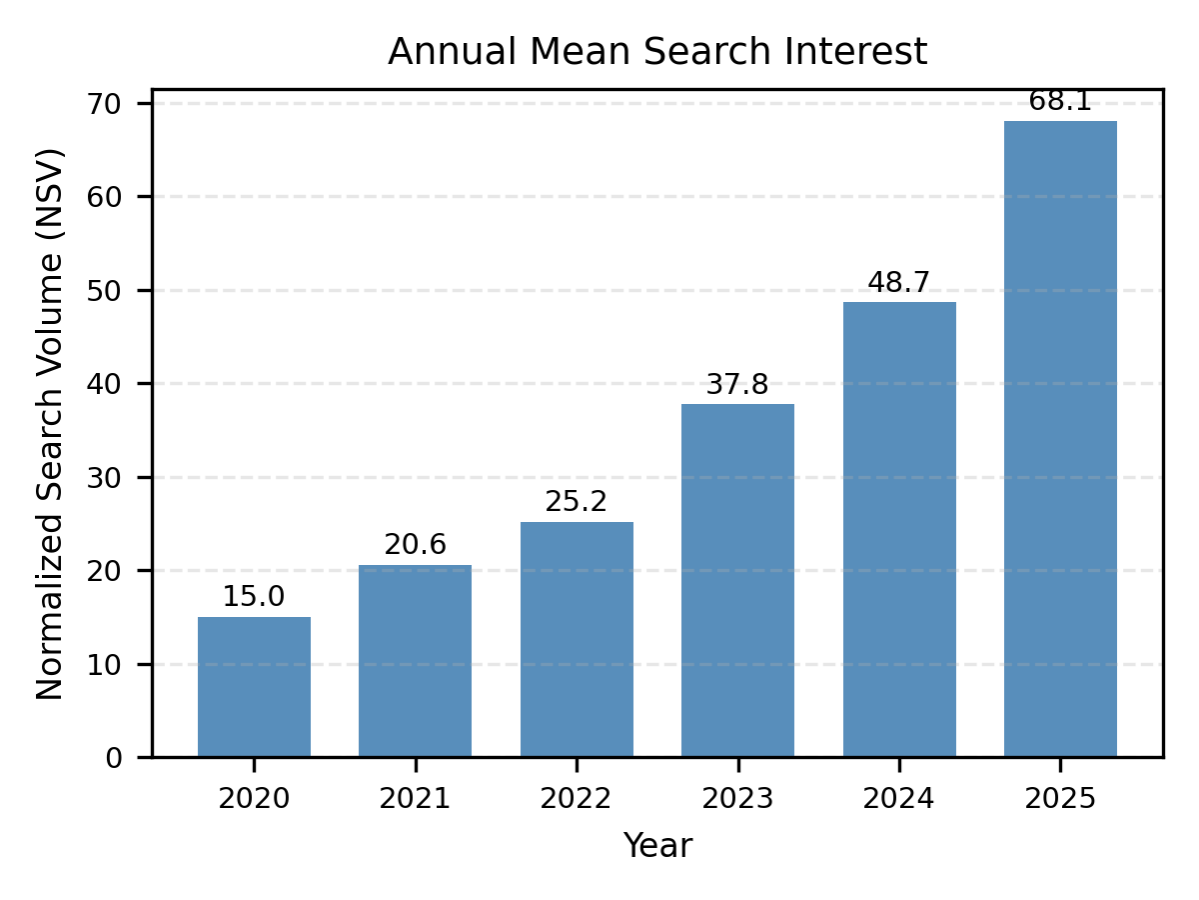
Annual growth of public interest in CO₂ laser treatments in Germany (2020‐2025). The chart illustrates the yearly mean normalized search volume (NSV) derived from the isolated STL trend component. Public information-seeking behavior demonstrated a continuous and statistically significant upward trajectory, growing from a baseline of 15.0 in 2020 to 68.1in 2025, representing a total sustained increase of 354%. Linear regression analysis confirmed a robust linear upward trend (slope=0.87 NSV/month; 95% CI 0.83‐0.92; R^2^=.96; *P*<.001), indicating a long-term shift in population-level interest.

Annual mean normalized search interest is summarized in Figure 3. The data demonstrate a continuous increase in public attention toward “CO2 laser” in Germany throughout the six-year observation period.

The annual average was lowest in 2020 (15.0) and increased incrementally in each subsequent year: 20.6 in 2021, 25.2 in 2022, 37.8 in 2023, 48.7 in 2024, and 68.1 in 2025. These absolute values correspond to year-over-year increases of 37% (2020‐2021), 22% (2021‐2022), 50% (2022‐2023), and 29% (2023‐2024). By 2025, search interest reached a total increase of 354% compared to the 2020 baseline.

To quantify the overall trajectory while accounting for seasonality, a linear regression was performed on the monthly trend component. The model estimated a monthly increase of 0.87 units (95% CI 0.83‐0.92; R^2^=.96; *P*<.001), confirming a robust and well-fitting linear upward trend over the multi-year period. These findings indicate that the rise in interest reflects a sustained long-term development in population-level information-seeking behavior rather than transient search spikes.

### Regional Trends

**Figure 4.**
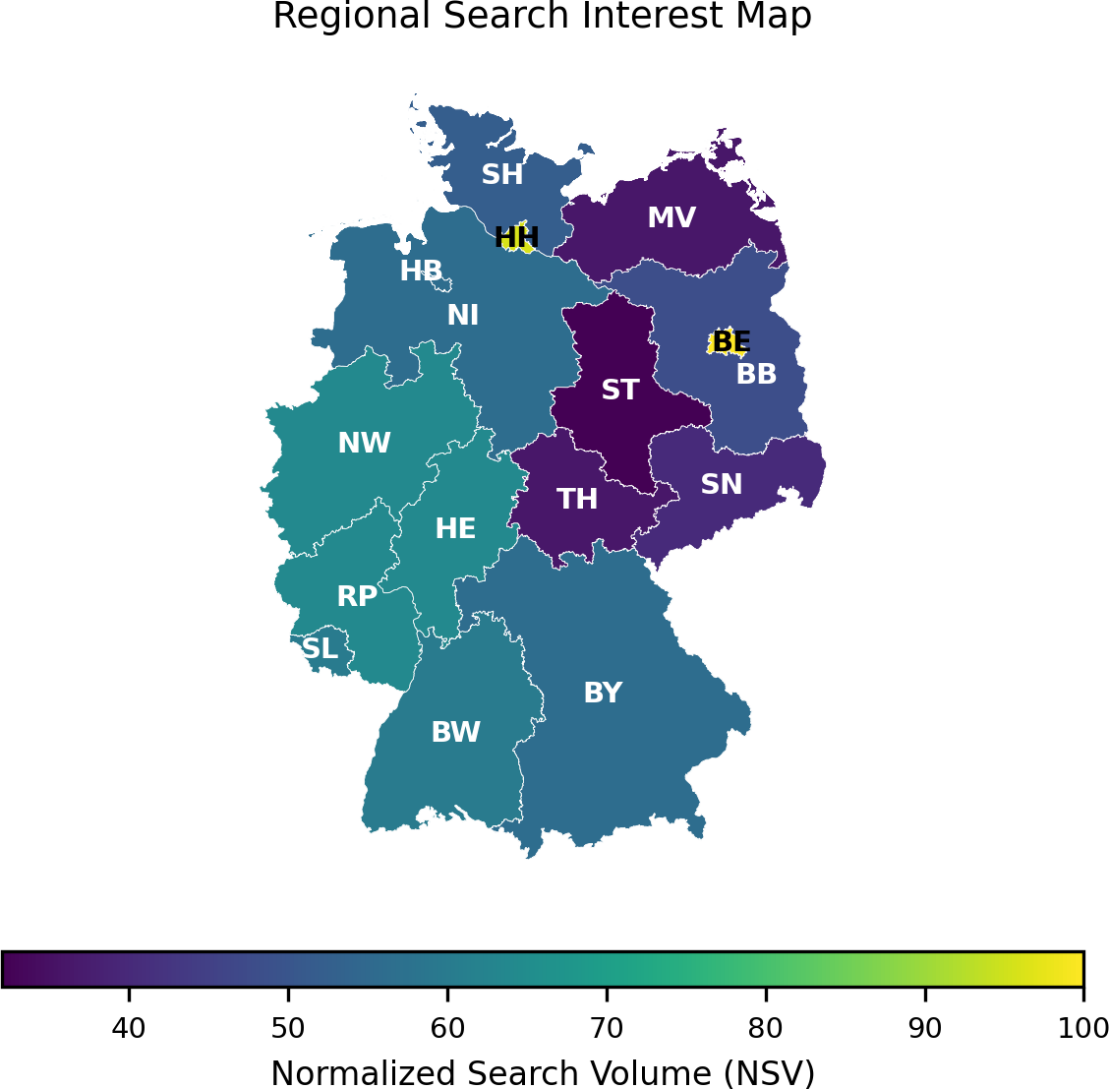
Geographic distribution of search interest for CO₂ laser treatments across German federal states. This choropleth map visualizes the average normalized search volume (NSV) 2020‐2025. The spatial data demonstrate a pronounced concentration of public interest in major metropolitan hubs. The highest search activity was observed in the city-states of Berlin (NSV=100) and Hamburg (NSV=96), followed by the populous states of Hesse (NSV=64) and North Rhine-Westphalia (NSV=64).

Analysis of state-level search interest for the 2020‐2025 period, revealed distinct regional concentrations of public attention toward CO2 laser treatments (Figure 4). The results indicate that search activity is most pronounced in major metropolitan regions. The highest search volumes were recorded in the city-states of Berlin (NSV 100) and Hamburg (NSV 96), followed by the populous states of Hesse (NSV 64) and North Rhine-Westphalia (NSV 64).

## Discussion

### Principal Findings

This study used Google Trends data for “CO₂ laser” in Germany (2020 – 2025) in the category „Health“ as a real-time proxy for public interest in this dermatologic procedure. Two robust patterns emerged: (1) a 354% sustained rise in the baseline trend component, increasing from a mean of 15.0 in 2020 to 68.1 in 2025 (Slope=0.87; 95% CI 0.83‐0.92; *P*<.001) and (2) a predictable seasonal rhythm peaking in winter and reaching its nadir in summer.

Search-based infodemiology has repeatedly been shown to mirror real-world utilization of esthetic interventions [10-12]. Our upward trend corroborates earlier global observations by Tijerina et al [12] who reported growing interest in CO₂-laser resurfacing over time [12]. The summer trough aligns with routine clinical practice, where CO₂-laser treatments are avoided during periods of high UV exposure because of pigmentary risk; this concordance further validates Google Trends as a behavioral signal. From an operational perspective, the yearly rebound that starts each September implies that public-facing education or advertising campaigns could be most effective when launched in late summer.

While an immediate surge in search interest might have been expected during the initial COVID-19 lockdowns in 2020, our data indicate a more sustained upward trajectory that intensified later in the study period. This temporal alignment provides a compelling contextual hypothesis for the „Zoom effect“. the theory that heightened self-scrutiny during frequent videoconferencing acts as a catalyst for esthetic interest.

Survey-based research supports this possibility, with over half of respondents attributing increased interest in cosmetic procedures to videoconferencing, and two-thirds reporting habitual self-observation on screen [10,11]. However, it is important to note that without a formal interrupted time series analysis to identify a specific step change, this link remains speculative. The observed growth likely reflects a complex interplay between the „Zoom effect“ and a broader, pre-existing trend toward minimally invasive esthetic treatments.

Social-media ecosystems likely amplify these dynamics. Platforms such as TikTok and Instagram serve simultaneously as marketing channels and informal information sources; their algorithmic curation immerses users in procedure-related content, reinforcing norms and sustaining interest [5]. Such amplification may intensify both the secular growth and the seasonal peaks identified here.

Further social media platforms increasingly function as both promotional channels and informal information sources for not only esthetic procedures. Algorithm-driven content delivery creates personalized environments where users are repeatedly exposed to procedure-related imagery, reinforcing interest and perceived norms. This algorithmic amplification may contribute to sustained search activity and intensify seasonal peaks observed in our data.

### Limitations

This study has several limitations inherent to the use of Google Trends data. First, the platform provides normalized search volumes without absolute user counts, limiting the ability to quantify population-level interest. Second, demographic details (eg, age, gender) are unavailable, restricting subgroup analysis. Most importantly, it is essential to distinguish between online search interest and actual clinical treatment uptake. The observed increase in search volume reflects heightened public curiosity and information-seeking behavior, rather than a direct measure of performed procedures.

### Conclusions

Google search behavior offers a reliable lens on public interest in CO₂-laser resurfacing, capturing both long-term growth and cyclical demand. This increasing digital curiosity heightens the need for rigorous practitioner training, regulatory oversight, and evidence-based patient education to ensure that online interest translates into safe, informed clinical decisions and mitigates potential complications.

## Abbreviations

CO_2_: carbon dioxide
LOESS: locally estimated scatterplot smoothing
NSV: normalized search volume
STL: seasonal-trend decomposition

## References

[1] Tierney EP, Hanke CW, Petersen J (2012). Ablative fractionated CO2 laser treatment of photoaging: a clinical and histologic study. Dermatol Surg.

[2] Zhang DD, Zhao WY, Fang QQ (2021). The efficacy of fractional CO_2_ laser in acne scar treatment: a meta-analysis. Dermatol Ther.

[3] Chen L, Xu H, Wang QY, Chen P, Wang LQ, Qin XM Treatment of surgical scars with fractional carbon dioxide (CO _2_ ) laser: a randomized controlled trial. Adv Wound Care.

[4] Taylor MA, Ituarte BE, Wysong A, Sulewski R, Wei EX (2024). Evaluating public interest in CO2 laser treatment: an analysis of Google Trends. J Cosmet Dermatol.

[5] Wojtara MS (2023). Use of social media for patient education in dermatology: narrative review. JMIR Dermatol.

[6] Eysenbach G (2009). Infodemiology and infoveillance: framework for an emerging set of public health informatics methods to analyze search, communication and publication behavior on the Internet. J Med Internet Res.

[7] Mavragani A, Ochoa G, Tsagarakis KP (2018). Assessing the methods, tools, and statistical approaches in Google Trends research: systematic review. J Med Internet Res.

[8] Mavragani A, Ochoa G (2019). Google Trends in infodemiology and infoveillance: methodology framework. JMIR Public Health Surveill.

[9] Sivesind TE, Szeto MD, Kim W, Dellavalle RP (2021). Google Trends in dermatology: scoping review of the literature. JMIR Dermatol.

[10] Shen CZ, Zhao AT (2025). The Influence of popular media on public interest in red-light therapy: longitudinal trend analysis. JMIR Dermatol.

[11] Motosko C, Zakhem G, Ho R, Saadeh P, Hazen A (2018). Using Google to trend patient interest in botulinum toxin and hyaluronic acid fillers. J Drugs Dermatol.

[12] Tijerina JD, Morrison SD, Nolan IT, Parham MJ, Nazerali R (2020). Predicting public interest in nonsurgical cosmetic procedures using Google Trends. Aesthet Surg J.

[13] Interesse im zeitlichen verlauf. Google Trends.

